# Correlation of Sonographic Classification of Neck Adenopathy (A-RADS) and Malignancy 

**DOI:** 10.22038/IJORL.2022.67255.3299

**Published:** 2023-01

**Authors:** Seyed Ali Alamdaran, Alieh Randian, Bashir Rasoulian, Amir Hossein Jafarian, Behzad Aminzadeh, Shabnam Niroumand

**Affiliations:** 1 *Department of Radiology, Faculty of Medicine, Mashhad University of Medical Sciences, Mashhad, Iran.*; 2 *Sinus and Surgical Endoscopic Research Center, Mashhad University of Medical Sciences, Mashhad, Iran. *; 3 *Pathology Cancer Molecular Research Center, Mashhad University of Medical Sciences, Mashhad, Iran.*; 4 *Department of Community Medicine, Faculty of Medicine, Mashhad University of Medical Sciences, Mashhad, Iran.*

**Keywords:** Adenopathy, Cancer, Lymph node, Ultrasound

## Abstract

**Introduction:**

Cervical adenopathy can be involved in various pathological processes. This study aimed to evaluate the ultrasound classification of cervical adenopathy (A-RADS) to choose the appropriate approach.

**Materials and Methods:**

This cross-sectional study was conducted among 294 patients with cervical adenopathy at Mashhad University of Medical Sciences during 2020-2021. The data of the long axis diameter, short axis diameter, shape, border, vascular pattern, presence of calcification and changes in cyst/necrosis, cortical echogenicity, hilum visibility, and location of involved lymph nodes were extracted. Lymph nodes was classified into four normal, reactive, suspicious & lymphoid disorders, and metastatic groups, based on ultrasound appearance (Adenopathy-reporting and data system). Diagnostic methods included follow-up, core needle biopsy (CNB), and fine needle aspiration (FNA), and surgical results. After determining the final diagnosis, demographic, sonographic, and pathological data were analyzed at a significance level of p<0.05.

**Results:**

Of 294 patients, 185 were benign, and 109 were malignant. There were no significant differences in the location, long axis diameter, shape, cystic or necrotic changes, calcification, and margins of the lymph nodes between the benign and malignant groups. The enlarged short axis diameter, invisible hilum with isoechoic cortex, and non-hilar vascularity were significantly higher in the malignant group (p<0.001). The malignancy rate was 8.7% in reactive cases, 48.5% in lymphoid disorders, and 90% in metastatic nodes.

**Conclusion:**

The results of this study shows that cervical lymph nodes can be classified based on short axis diameter, cortex and hilum echo-texture and vascular pattern into normal, reactive, suspicious & lymphoid disorders, and metastatic, which have a high concordance with pathologic results.

## Introduction

Approximately one-third of the body's lymph nodes are in the neck region (1). Cervical adenopathy can be seen in various pathological processes such as lymphoma, tuberculosis, and metastasis (2). The importance of adenopathy is that it reduces the 5-year survival of patients with metastatic lymph nodes. Therefore, accurate differentiation between these conditions is crucial to choosing the appropriate treatment and assessing the prognosis (3,4).

Different imaging modalities such as CT, MRI, and ultrasound have been used to evaluate cervical lymph nodes (3,5). Although some studies have not reported any significant difference between these modalities in cervical lymph node assessment, other studies have mentioned ultrasonography as the most sensitive technique (4,6). Ultrasound can detect small lymph nodes (under 10 mm) better than other techniques. In addition, it can evaluate both internal and external anatomy, number, size, shape, and margins of cervical lymph nodes (6-8). Also, intra-nodal vascular patterns, blood flow velocity, and vascular resistance can be evaluated by Doppler ultrasound (9). Cervical ultrasound has been of interest more than other diagnostic approaches, especially as the first line. Ultrasound is associated with advantages such as safety, being fast, low cost, availability, no need for special medical facilities, and the possibility of performing diagnostic procedures. Numerous studies have been conducted on the relationship between sonographic parameters such as size, shape, margin condition, and vascularity of cervical lymph nodes and cervical adenopathy causes (10-12). However, different criteria have been proposed to distinguish malignant from benign lymph nodes. According to the different diagnostic approaches for various types of lymphadenopathies, their classification is valuable to choosing the appropriate diagnostic approach, reducing the costs and duration of diagnosis, and improving diagnostic-therapeutic outcomes. This study aimed to evaluate the ultrasound classification of cervical adenopathy to choose the appropriate diagnostic method and match the pathology results. 

## Materials and Methods

This study was conducted on 294 patients with cervical adenopathy who were referred to a clinic affiliated with Omid Hospital of Medical Sciences Mashhad University during 2020-2021 after obtaining the Code of Ethics Committee (IR. mums. medical. Rec. 1400.257). The patients with cervical adenopathy without known cancer or patients referred for the staging of known cancer were included in the study. Patients with reactive lymph nodes caused by thyroiditis and acute infections and patients whose follow-up, cytology, or pathology results were not available were excluded from the study. 

Gray scale and Doppler ultrasound were performed using an Esoate-class C machine with a 16-8 MHz probe by a single examiner experienced in head and neck sonography.

Ultrasound variables such as location, short axis diameter, long axis diameter, shape, border, cortical echogenicity, visibility of the echogenic hilum, cortex echogenicity, existence of necrotic/cystic changes, calcification, and vascular pattern of lymph nodes were recorded. Normal cervical lymph nodes are oval with echogenic hilum and symmetrical cortex and hilar blood flow, the largest of which is one or two prominent lymph nodes in the jugulo-digastric area with a short axis diameter (SAD) about 1 centimeter and smaller. In other cervical levels, lymph nodes are not prominent. Sometimes one slightly larger lymph node is observed at the junction of the thoracic duct and subclavian vein in the left fourth level of the neck, although it is smaller than normal lymph nodes of the jugulo-digastric region. A prominent enlarged lymph node outside this pattern was considered adenopathy, especially in levels 3, 4, 5, and 6. In terms of shape, the largest short and long axes of lymph nodes were measured. The shape index was calculated as the ratio of the short and long axes. As a result, lymph nodes with an index below and above 0.5 are considered oval and round, respectively (2,3,5). The cortical echogenicity of lymph nodes is divided into normal hypo-echogenicity and increased iso-echogenicity. Normal or reactive lymph nodes had symmetric hypoechoic cortex and hyperechoic hilum (normal cortico-medullary echotexture). Abnormal adenopathies are associated with small or invisible hilum, isoechoic cortex with hilum (absent cortico-medullary differentiation), sometimes cystic changes and calcification. The criterion for classifying the cortical echogenicity of adenopathy was a comparison with hilum. According to the border, lymph nodes are divided into sharp and indistinct. Doppler examination was performed in suspected adenopathies. The vascular pattern is defined as the feeding arteries’ branching pattern, divided into hilar, non-hilar, and decreased vascularity. The non-hilar vascularity and enlarged lymph node with decreased vascularity are abnormal. Lymph nodes with a enlarged short axis diameter compared to other cervical lymph nodes, abnormal cortico-medullary echotexture, and abnormal vascularity were considered adenopathy. According to the differ these criteria, we classified lymph nodes into four normal, reactive, suspicious & lymphoid disorders, and metastatic groups based on ultrasound appearance (adenopathy-report and data system): reactive lymph nodes are an enlarged oval shape with visible hilum and increased hilar vascularity. Suspicious & lymphoid disorders lymph nodes are enlarged with small or invisible hilum and hypoechoic cortex, sometimes with intra-nodal reticulation and conglomeration or necrotic changes. Various types of vascularity are seen in this group. The metastatic adenopathies have invisible hilum with increased echogenicity of cortex (isoechoic cortex with hilum) and sometimes with cystic and calcification changes. The non-hilar vascularity and decreased vascularity within the enlarged lymph node are seen in the metastatic adenopathies. 

**Fig 1:**
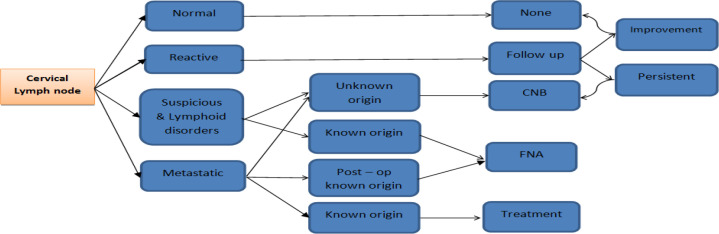
Diagnostic approach in cervical adenopathy in this study

Four diagnostic approaches of follow-up, FNA (fine needle aspiration), CNB (core needle biopsy), and therapeutic neck dissection, were used for patients: In reactive adenopathies, patients were treated medically with Co-amoxiclav for two weeks. After the follow-up ultrasound was done 3-4 weeks later, if adenopathies improved, it was called reactive, and if it was stable or increased in size, they underwent core needle biopsy. Suspicious & lymphoid disorders and metastatic lymph nodes with an unknown origin were subjected to core needle biopsy. CNB was mainly performed with 14 or 16-gauge needles. For greater safety, the needle trajectory was chosen from the lateral side to the medial side of the neck. The lateral to medial trajectory chosen provides better needle visualization, avoids vessels or viscera, and better post-op compression, thereby preventing the possibility of complication. CNB aims to check the immuno-histo-chemical (IHC) or PCR samples to achieve a definite diagnosis. Metastatic adenopathy in known cancer patients underwent therapeutic neck dissection. In some patients with known cancer and suspicious adenopathy, FNAC was performed to differentiate reactive from metastatic lymph nodes determining the extent of neck dissection. Figure 1 summarizes our diagnostic approach in cervical adenopathy. 

The investigated lymph nodes were divided into benign and malignant types based on cytopathology results and follow-up results. Finally, the sonographic characteristics of the lymph nodes were compared with the final diagnosis results (cytopathology or follow-up outcome). After determining the final diagnosis, demographic, sonographic and pathological data were analyzed with SPSS version 21 software at a significance level of p<0.05 for the Chi-square test and T-test. 

## Results

In 294 patients referred with cervical adenopathy, four diagnostic approaches, including follow-up in (11.5%) 34, FNAC in (38.7%) 114, CNB in (43.5%) 128, and therapeutic neck dissection in (6%) 18 patients were used. The final diagnosis was as follows: 185 patients were malignant, and 109 were benign. The average age of patients with benign causes was about ten years less than that of patients with malignant causes (37.7 ± 18.3 years versus 46.8 ± 16 years, p<0.265).In comparison between benign and malignant groups, the results showed that cervical adenopathies are more common in women: 67% of the benign group and 60.5% of the malignant group were females (p=3180). Although half of benign (reactive) adenopathies are located at the neck’s first and second levels, the adenopathy’s location has no value in differentiating malignant and benign causes (p=0.111). There was no difference in the involvement side in the two groups of patients (p=0.115). Table 1 shows the demographic data of our patients. The morphologic characteristics of abnormal lymph nodes were evaluated (Table 2). The comparison between the short axis and long axis diameter in two groups of benign and malignant patients showed that the short axis in the malignant group was significantly larger than the benign group (p=0.000), but the long axis difference was insignificant. The shape of the lymph nodes and the state of their margins; no significant difference was reported between the benign and malignant groups (p= 0.785 and p= 0.558, respectively) in Figure 1.

**Table 1:** Demographic data of examined patients

**Parameters**				**Benign N (%)**	**Malignant N (%)**	**P-value**
Disease				(38.0)109	(62)185	0.31
Age				37.7±18.3	46.8±16	0.00
Gender		Male		73 (39.5)	112(60.5)	0.38
		Female		36(33.0)	(67.0)73	
Side	Right			**40(38.1)**	**88 (50.6)**	0.11
	Left			**40(38.1)**	56 (32.2)	
	Bilateral			25 (28.3)	30 (54/5)	
				

**Table 2:** The ultrasound morphologic characteristics of cervical adenopathy

**Variables**		**Tumor type / Number** **(%)**			**P value**		
		**Benign**	**Malignant**				
Size	Long axis	106 (23mm)	181 (26mm)		**0.009**	
	Short axis	106 (11mm)	181 (13mm)		**0.000**	
Shape	Oval	92(95)	133(94)			**0/785**
	Round	5(5)	9(6)			
Margin	Obvious	92(95)	134(94)			**0/558**
	**Unclear**	**5(5)**	**8(6)**			
Hilum	Present	60(62/5)	39(25)			**0/000**
	Absent	36(37/5)	119(75)			
Homogeneity	Homogeneity	**82(84/5)**	57(40)			**0/000**
	Heterogeneity	15 (15/5)	85(60)			
Cortical Echogenicity	**Hypoechoic**	**89(92)**	**104(67)**			**0/000**
	**Isoechoic**	**8(8)**	**37(24)**			
	**Mixed**	**0(0)**	**15(10)**			
Echo-texture	**Normal**	**30(30)**	**2(1)**			**0/000**
	**Abnormal**	**70(70)**	**165(99)**			
Cyst/necrosis	**Present**	**11(12)**	**37(25)**			**0/007**
	**Absent**	**84(88)**	**110(75)**			
Calcification	**Present**	**15(16)**	23(16)			0/556
	**Absent**	80(84)	124(84)			
Vascularity	**Hilar**	**52(67)**	**16(11)**			**0/000**
	**Non-hilar**	**11(14)**	**102(72)**			
	**Decreased**	**15(19)**	**24(17)**			

The absence of hilum was reported in 75% of the malignant group and 37.5% of the benign group, with a statistically significant difference (p=0.000). Abnormal cortico-medullary echotexture (the small or invisible hilum with iso-echoic cortex or reticulation) was abnormal in 99% of patients in the malignant group, which was significantly higher than benign group (70%) (p=0.000). 

The frequency of the isochoic or mixed-echoic cortex in the malignant group was 24% and 10%, respectively, which was significantly higher than the benign group with a frequency of 8% and 0% (p=0.000). Therefore, the heterogeneity of adenopathies in the malignant group with a significant difference was more common (60% vs. 15.5%, p=0.000). Also, non-hilar vascularity was significantly more reported in malignant group patients: 72% versus 14%, p=0.000). Cystic or necrotic changes were more common in the malignant group than in another (25% vs. 12%, p=0.007). 

In terms of the frequency of calcification, no significant difference was reported between the two groups (p=0.556). 

Abnormal cortico-medullary echo-texture (the small or invisible hilum or/and isoechoic cortex or reticulation) had 98.8% sensitivity, 30% specificity, 70.21% positive predictive value, and 93.75% negative predictive value in the diagnosis of malignancy. Considering all the variables, the sensitivity of ultrasound in diagnosing malignant lymph nodes was 74.69%, its specificity was 92.16%, positive predictive value was 97.83%, and its negative predictive value was 43.52%. 

Among malignant cases, 34 cases were diagnosed with lymphoma, and 151 were diagnosed with metastatic adenopathy from the skin, thyroid, salivary glands, larynx, nasopharynx, tongue, and breast. Among the benign adenopathy, 16 cases were granulomatous (mainly mycobacterial infections), and the rest were reactive lymphadenitis, including lymphoid hyperplasia, non-necrotizing granulomatosis, necrotizing lymphadenitis (Kikuchi disease), foreign body reaction, Rosai-dorfman disease. 

**Fig 2:**
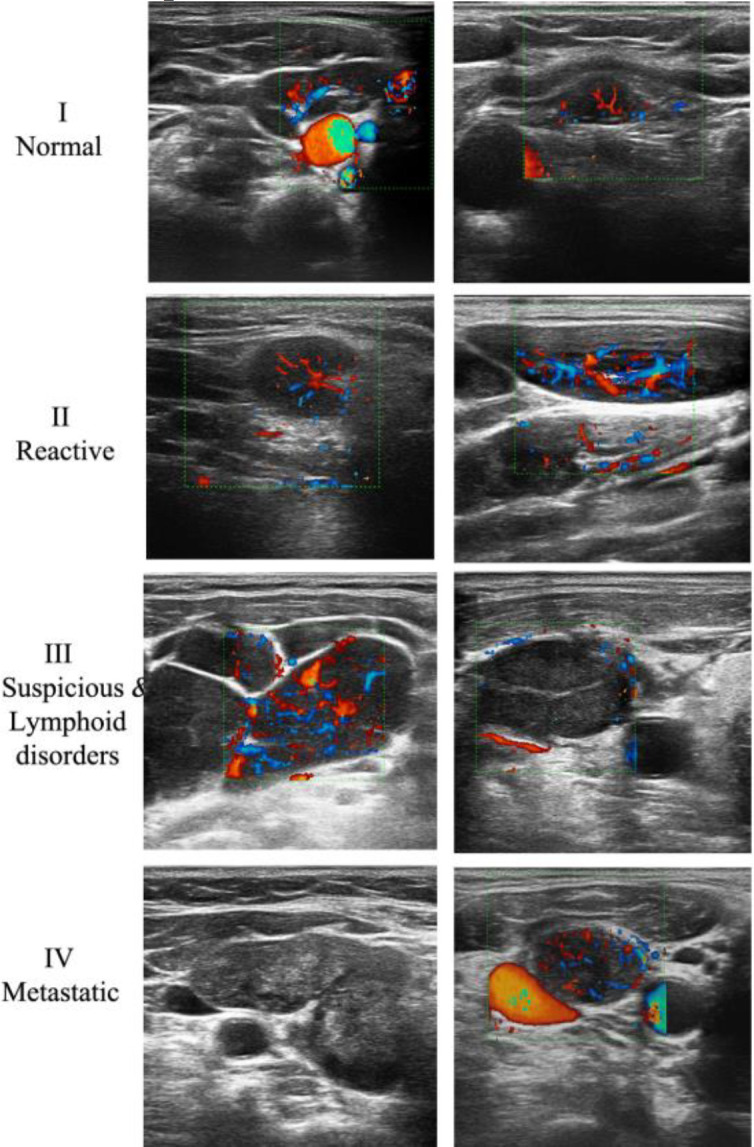
Adenopathy Reporting and Data Systems classification in ultrasound images; the ultrasound appearance of normal lymph nodes (I) and adenopathies (II-IV) according to the proposed categories (A-RADS): In group I, it shows normal lymph nodes in the jugulo-digastric area (right) and other cervical levels (left). In group II, it shows short axis (right) and long axis (left) of reactive adenopathies with increased hilar hyper-vascularity. In group III, it shows huge hypoechoic adenopathies of non-Hodgkin lymphoma (right) and necrotizing granulomatous lymphadenitis with decreased vascularity (left). In group IV, it shows adenopathy with iso-echoic cortex (right) or peripheral non-hilar vessels (left) in two different cases with squamous cell carcinoma

As mentioned, lymph nodes were classified into four normal, reactive, suspicious & lymphoid disorders, and metastatic groups based on ultrasound appearance: Reactive lymph nodes have an oval shape, normal echo texture, and hilar vascularity. Metastatic adenopathies have invisible hilum and increased cortical echogenicity, non-hilar vascularity, and sometimes cystic changes or calcification. Adenopathies suspected of lymphoid disorders are large and have small or invisible hilum and hypoechoic cortex, sometimes with intra-nodal reticulation, conglomeration, or necrotic changes (Figure 2). The malignancy rate was 8.7% in reactive adenopathy, 48.5% in lymphoid disorders adenopathy, and 90% in metastatic adenopathy. Table 3 shows the ultrasound categories, pathologic diagnosis, and risk of malignancy in neck adenopathies. 

**Table 3:** Ultrasound categories, pathologic diagnosis, and risk of malignancy in neck adenopathies in our study

**Ultrasound categories**	**Number (%)**	**Pathologic diagnosis**	**Number (%)**	**Risk of malignancy (%)**
Reactive	46 (15.6)	Reactive	40 (87)	<10%
		Metastatic	4 (8.7)	
		Granulomatosis	2 (4.3)	
Suspicious & Lymphoid disorders	99 (33.7)	Reactive	40 (40.5)	≈50%
		Lymphoma	34 (34.3)	
		Metastatic	14 (14.1)	
		Granulomatosis	11 (11.1)	
Metastatic	149 (50.7)	Metastatic	132 (88.6)	90%
		Reactive	12 (8)	
		Granulomatosis	3 (2)	
		Lymphoma	2 (1.3)	

## Discussion

Lymph node metastasis is common in head and neck malignancy (12). Various studies have investigated ultrasound findings in differentiating between benign and malignant adenopathy. Although some are valuable in predicting malignant and benign adenopathy, studies have shown that none of these ultrasound findings alone can differentiate benign adenopathy from malignant ones. 

Normally, there are one or two prominent lymph nodes in the jugulodigastric area with a short axis of about one centimeter. In other cervical levels, there is no central cervical lymph node. A larger-than-expected short axis compared to other lymph nodes was considered adenopathy. This criterion helps diagnose cervical adenopathy and prevent over-diagnosis of normal cervical lymph nodes as adenopathy. Our results showed that the mean short and long axis diameters of the lymph nodes in the malignant and benign groups are close to each other with a difference of 2-3 mm, which is consistent with the results of similar studies (13-15). However, the criterion of lymph node size has a significant pitfall in the diagnosis of suspicious lymph nodes; with a size of 11 mm for level 2 and 10 mm for other levels, this criterion has one-third pitfall and sizes with less maximum (6 mm) have a maximum negative predictive value of 90% (12). According to LO et al.'s study, the size of lymph nodes is not an important criterion in diagnosing lymph nodes suspected of malignancy (15). 

Although in various studies, malignant lymph nodes often have a round shape, while benign lymph nodes are oval (4,16,17), in our study, most of the lymph nodes in the benign and malignant groups were oval, and the shape criterion has no diagnostic value in differentiating benign from malignant adenopathy (p=0.785). In the study of Dangore-Khasbage et al., it was not an important criterion for differentiating benign from malignant adenopathy, as it was in our study (18).

In the study of Ryu KH et al., a non-circumscribed margin has a diagnostic value in diagnosing malignant lymph nodes (16). However, in a study by Lakshmi et al., 100% of malignant lymph nodes had sharp borders (17). However, our study and Abhishek Gupta et al. showed that most benign and malignant lymph nodes have clear margins, the margin parameter is an unreliable criterion in differentiating adenopathy, and most malignant lymph nodes have clear margins (19). In studies, most malignant adenopathy lacks echogenic hilum, and most benign adenopathy has central echogenic hilum (4, 16-18, 20). In our study, lack of echogenicity was significantly associated with malignancy (p=000). However, Ahuja and Ying stated that the central hilum might not be seen in small benign lymph nodes (6, 21). Although most malignant lymph nodes, especially lymphoid disorders, have a hypoechoic appearance, in our study, Ryu KH and Lakshmi's studies, hyper-echogenicity is associated with malignancy (16,17). According to the results of this study, echogenicity is an important criterion in differentiating benign from malignant adenopathy. 

Lo et al. and King et al. stated that cyst/necrotic changes are a reliable criterion in differentiating between benign and malignant lymph nodes (15,22). Also, in Ryu KH's and Lakshmi's studies, the presence of necrosis is significantly related to the possibility of lymph node malignancy (16,17). In the study of Van den Brekel et al., necrosis is a valuable criterion in diagnosing malignant adenopathy, and the combination of this criterion with the diameter increases the sensitivity of the diameter (13). However, the results of our study showed that necrotic changes are also observed in benign adenopathies such as mycobacterial infections, and its significant relationship with malignancy was not observed (p=0.007). 

While in the study of Ryu KH et al., calcification is a reliable criterion in differentiating malignant adenopathy (19.3% vs. 3.8%) (16), but in our study, the presence of calcification has no diagnostic value in differentiating benign from malignant lymph nodes (p=0.556), and it is also seen in reactive and mycobacterial lymphadenitis.

In this study, lymph nodes with echogenic hilum and symmetric hypoechoic cortex were classified as normal or reactive, and adenopathies with small or invisible hilum, increased cortical echogenicity or reticulation, sometimes with necrotic changes and calcification were classified as suspicious & lymphoid disorders or metastatic. Although in the study conducted by Imani M and colleagues (20) and in the study conducted by Gupta A et al., most benign and malignant lymph nodes were homogeneous, this criterion was not useful for differentiating types of adenopathy (2). However, in our study, small or invisible hilum with cortical iso-echogenicity or reticulation (abnormal echo texture) is an important criterion in differentiating between benign and malignant adenopathy, especially in metastatic cases. 

Dangore-Khasbage et al. stated that hilar vascular pattern is a predictive factor for benign adenopathy (18). In the study conducted by Ryu KH et al., and several other studies, mixed and peripheral vascularity was associated with malignancy (9,16). Also, in Gupta A et al.'s study, peripheral vascularity was more common in malignant lymph nodes (2). In our study, the non-hilar (peripheral) vascular flow was significantly higher in malignant adenopathy.

Various studies showed the high diagnostic value of ultrasound in differentiating cervical adenopathy. In examining the diagnostic value of neck ultrasound in differentiating benign from malignant lesions, Jayapal et al. showed that the true positive value was 30.5%, the false positive value was 11.5%, the false negative value was 10.5%, and true negative value was 47.5%. The ultrasound result was associated with 73.81% sensitivity, 80.67% specificity, and 77.83% overall accuracy (23). In a study by Kamat Rohan et al., the results showed that neck ultrasound has a sensitivity of 97% (10). In another study, ultrasound was associated with 83.3% accuracy and 82.4% specificity in diagnosing metastatic lymph nodes in patients with head and neck malignancy (11). In our study, assessment of ultrasound diagnostic value shows that sensitivity and specificity for ultrasound diagnosis of lymph nodes are 74.69% and 92.16%, respectively, and these numbers are suitable. 

According to the different diagnostic approaches, adenopathies were classified based on ultrasound appearance into three reactive, suspicious & lymphoid disorders and metastatic: Reactive lymph nodes are enlarged (SAD>1cm), hypoechoic, visible hilum, and increased hilar vessels, metastatic adenopathies have a iso-echoic cortex, invisible hilum, decreased or non-hilar vessels, sometimes cystic changes or calcification, and adenopathies of suspicious & lymphoid disorders are hypoechoic, enlarged (SAD>1cm), small or invisible hilum occasionally with intra-nodal reticulation and conglomeration. Different types of vascularity patterns are seen in suspicious & lymphoid disorders. Since the majority (85.9%) of group III patients included adenopathies related to reactive, granulomatous, and lymphoma, we chose the name suspicious & lymphoid disorders for it, although a number of metastatic adenopathies have a similar ultrasound appearance and are included in this group. Respectively four diagnostic approaches, including follow-up, CNB, FNA, and therapeutic neck dissection, were used for patients. 

FNA has a known value to confirm the presence of metastasis in lymph nodes and is used to differentiate between reactive and metastatic adenopathy. It does not have a high value in determining the nature of the cause of adenopathies, especially in cases of lymphoid disorders such as lymphoma and mycobacterial adenitis. CNB is used to diagnose the cause of adenopathy with the help of IHC and PCR studies. Therefore, in this group of patients, we used CNB for immuno-histochemical and/or PCR studies which have high diagnostic accuracy in determining the nature and origin of adenopathies (19). Suspicious & lymphoid disorders or metastatic adenopathies with an unknown origin were subjected to core needle biopsy. CNB was mainly performed with 14 gauge needles in lymphoid disorders and 16 gauge needles in the metastatic group. Overall, using the appropriate approach, in addition to high accuracy in reaching the diagnosis, avoids additional procedures and wasting time. 

Table 4 shows the proposed ultrasound categories, management system, and risk of malignancy in cervical lymph nodes. 

Figure 2 shows the ultrasound appearance of normal lymph nodes and adenopathies according to the proposed categories (A-RADS).

**Table 4:** Proposed ultrasound classification and diagnostic approach of neck adenopathy

**Ultrasound categories (A-RADS)**		**Ultrasound ** **appearance**		**Management**
I	Normal	SAD<1cm with few hilar vessels		None
II	Reactive	SAD=1-1.5cm, hypoechoic cortex with visible hilum and increased hilar vessels		Follow up
III	Suspicious & Lymphoid disorders	SAD>1cm, hypoechoic cortex with small or invisible hilum. Hilar, non-hilar or decreased vascularity	Unknown origin	CNB
			Known origin	FNA
IV	Metastatic	Iso-echoic cortex and invisible hilum with non-hilar vessels	Unknown origin	CNB
			Known origin	Treatment
			Post -op of known origin	FNA
Normally, there are 1-2 prominent lymph nodes in the Jugulo-digastric area with SAD ≈ 10 mm. Others lymph nodes are small, except one in the supraclavicular area. An unexpected prominent lymph node out of this pattern is abnormal. Occasionally, intra-nodal reticulation, conglomeration and necrotic changes in lymphoid disorders and cystic changes in metastatic nodes are seen. Follow up was done for 1 month. if adenopathy improved, it was called reactive, and if it was stable or increased in size, changed into category III. FNA is used to differentiate metastatic and reactive adenopathy, and CNB is used to diagnose the cause of adenopathy with the help of IHC and PCR. CNB was mainly performed with 14 gauge needles in suspicious lymphoid disorders and 16 gauge needles in the suspicious metastatic group. Therapeutic neck dissection with primary tumor resection. Occasionally, there are special therapeutic protocols for some tumours, such as nasopharyngeal carcinoma.				

According to the results of this study, there is a high concordance between pathological results and ultrasound appearance, which can be used in choosing the appropriate treatment approach. However, this is a preliminary study; more studies are needed to check its value and provide the necessary corrections. 

Although the criterion of cortical thickness in axillary adenopathies has a decisive role in dealing with breast cancer patients (14), usage of A-RADS for other superficial adenopathies (axillary and inguinal) is valuable in clinical practice, and it also needs further studies.

## Conclusion

The enlarged short axis diameter, absence of hilum, iso-echogenicity of the cortex, and non-hilar vascularity in the malignant group were significantly higher than the benign group on ultrasound. 

The results of this study show that cervical lymph nodes can be classified based on short axis diameter, cortex and hilum echo-pattern and vascular pattern into four normal, reactive, suspicious & lymphoid disorders, and metastatic (A-RADS), which helps in choosing the next diagnostic approach. 
